# An Account of the New Process for Embalming, and of Preserving Anatomical Preparations, &c.

**Published:** 1841-04-01

**Authors:** 


### AN ACCOUNT OF THE NEW PROCESS FOR EMBALMING, AND OF PRESERVING ANATOMICAL PREPARATIONS, SPECIMENS OF NATURAL HISTORY, &c. Translated from the French of *M. Gannal*. By *R. Harlan*, M.D. Octavo, pp. 264. Philadelphia, 1840.

NEARLY one-half of the present volume is dedicated to an historical survey of the art of embalming among the ancients, especially the Egyptians, and more than half of the remainder is taken up with the modern processes employed for preserving the lifeless human frame, which ought to be consigned to the grave or the flames. Over both these portions of the book we shall pass entirely, as quite useless (the first particularly) in a practical point of view. What care we whether the Egyptians pitched and bandaged up the stinking carcases of their friends, to preserve them for forty thousand years, till the spirit returned to animate the black mummy—or because the soul kept possession of the clay for a thousand years after death—or because they were unable to bury the dead during the periods of inundation—or to secure the remains of their defunct relations from the voracity of animals —or from motives of filial or parental affection—or, finally, to furnish important remedies against dangerous diseases—a mummy being a sovereign cure for various maladies.

Whatever was the motive for embalming human bodies, the practice was absurd, as well as superstitious. If it became universal and continued for a million or two of years, the earth would be covered with mummies, instead of living beings! Sepulture, indeed, is only an inferior evil of the same kind. Burning the dead, and thus resolving at once the constituents of the cadaver into its original elements, is the most philosophic process, and that which would entail less mischief, as well as expense, on the living survivors.

We see that M. Gannal has reserved for his own profit the secret of embalming bodies so as to keep them in a state as nearly as possible resembling life. We quarrel not with him for this piece of charlatannerie, since he has disclosed the process for preserving anatomical preparations. This is all we want with the whole "art and mystery" of embalming.

M. Gannal's memoir was presented to the Institute on the 4th of March, 1838, and a commission was appointed to examine and test it.

Meantime M. Serres placed a corpse at M. Gannal's disposal in La Pitié, which he bathed in a tub containing two pails of alum, two of common salt, and one of nitrate of potash. The subject appeared to be well preserved, on repeated examinations. At the end of six weeks it was opened, when the flesh and viscera were found in a state of preservation. On the 12th November, 1834, M. Orfila placed two bodies under our author's management. These were bathed in a similar liquid at ten degrees.* On the 2d December the commission examined them, and they were consigned to dissection. On the same day another subject was injected with eight quarts of the saline solution at ten degrees. At the end of December these three subjects were examined, and found to be in good state of preservation; but it was remarked that the skin as well as the flesh had assumed slight appearances of decay, in respect to consistence and colour. The deep-seated organs remained nearly natural.

A commission named by the Academy examined these subjects, and demanded new experiments.

" Here it may be remarked that it required double the quantity of fatty matter for this, than for a fresh subject, and that the most delicate arterial net-work had been prepared by the injection.

These experiments, which lasted for half the month of May, satisfied me that an injection of ten or twelve degrees of density, and immersion of the body in a bath of the same liquid, will suffice for a preparation destined for ordinary anatomical purposes, and will allow of dissection after several months." 207.

The following extract from the second report of the Commissioners contains all that is necessary for insertion in this article :—

" From the series of experiments which we have just exposed, it results:

1. That a solution of alum, of salt, and of nitrate of potash, injected at ten degrees, answers for preserving bodies at a temperature below ten degrees of the thermometer; that, for a more elevated temperature, it is necessary to carry the density to twenty-five or thirty degrees, and immerse the subject in a liquid of ten or twelve degrees.

2. That it is preferable to employ the acetate of alumine, because it preserves better; as the skin experiences no alteration, and as the central organs remain *natural*, excepting the colour of the muscles which become bleached.

3. That the chloride of aluminium offers the same advantages.

4. That, in order to preserve parts of bodies which have not been injected, it is necessary to immerse them in a mixture of water, and of the acetate or chloride, marking five or six degrees.

But this part of the operation is transferred to the experiments which are to be undertaken on the preservation of objects of pathological anatomy.

Gentlemen, such are the series of experiments made by M. Gannal, since the first provisionary report was presented to you.

The commission has attentively followed the new experiments; the results obtained, demonstrate that by M. Gannal's process bodies for dissection may be preserved, and the preservation prolonged beyond the term exacted by the most minute investigations.

---

* What does he mean by ten degrees?

As we have already stated, the soluble salts with an aluminous base, offer this preservative method, without any danger in their use, and they can also be procured at a low price.

Their antiseptic properties are founded on their chemical action, which modifies animal substances either by depriving them of their water of composition, which determines their putrefaction, or in opposing themselves to its immediate action.

It is, then, only an act of justice rendered to M. Gannal, in considering his labour as an important service rendered to science and to humanity, and which may prove of great utility in anatomical explorations, and in legal medicine." 231.

Dr. Harlan, the translator, bears testimony, from occasional observation to the merits of M. Gannal's process; but thinks that, "he has, perhaps, over-rated the extent and importance of his discovery." This will, doubtless, be found to be the case, and it applies to all new discoveries and inventors.

